# The herbal extract EPs® 7630 increases the antimicrobial airway defense through monocyte-dependent induction of IL-22 in T cells

**DOI:** 10.1007/s00109-020-01970-3

**Published:** 2020-09-19

**Authors:** Katrin Witte, Egon Koch, Hans-Dieter Volk, Kerstin Wolk, Robert Sabat

**Affiliations:** 1 Interdisciplinary Group of Molecular Immunopathology Dermatology/Medical Immunology, Charité - Universitätsmedizin Berlin, Berlin, Germany; 2 BIH Center for Regenerative Therapies, Charité - Universitätsmedizin, Berlin, Germany; 3 Dr. Willmar Schwabe GmbH & Co. KG, Karlsruhe, Germany; 4 Present Address: Karlsruhe, Germany; 5 Institute of Medical Immunology, Charité - Universitätsmedizin Berlin, Berlin, Germany; 6 Psoriasis Research and Treatment Center, Charité - Universitätsmedizin Berlin, Berlin, Germany

**Keywords:** *Pelargonium sidoides*, Antimicrobial proteins, IL-22, IL-17, T cell, Monocyte, NKT cell

## Abstract

**Abstract:**

The phytotherapeutic compound EPs® 7630, an extract manufactured from *Pelargonium sidoides* roots, is frequently used for the treatment of airway infections. Nevertheless, the knowledge of the mode of action of EPs® 7630 is still sparse. Our study aimed at further elucidating the underlying pharmacological mechanisms by focusing on antimicrobial defense mechanisms of EPs® 7630. While investigating the influence of EPs® 7630 on lymphokine production by PBMCs, we found that EPs® 7630 is a novel inducer of IL-22 and IL-17. This cytokine-inducing effect was most pronounced for IL-22 and clearly dose-dependent starting from 1 μg/ml of the extract. Furthermore, EPs® 7630 pretreatment selectively enhanced the IL-22 and IL-17 production capacity of CD3/28-activated PBMCs while strongly limiting the IFN-γ production capacity of innate lymphoid cells. The relevance of EPs® 7630–induced IL-22 production was proven in vitro and in vivo*,* where IL-22 provoked a strong increase of the antimicrobial protein S100A9 in lung epithelial cells and pulmonary tissue, respectively. A detailed analysis of IL-22 induction modi revealed no direct influence of EPs® 7630 on the basal or anti-CD3/CD28 antibody-induced IL-22 production by CD4^+^ memory T cells. In fact, EPs® 7630–induced IL-22 production by CD4^+^ memory T cells was found to be essentially dependent on soluble mediators (IL-1/IL-23) as well as on direct cellular contact with monocytes. In summary, our study reveals a new immune-modulating function of EPs® 7630 that might confer IL-22 and IL-17-induced protection from bacterial airway infection.

**Key messages:**

- EPs® 7630 selectively strengthens IL-22 and IL-17 production of memory T cells.
- EPs® 7630 limits the IFN-y production capacity of innate lymphoid cells.
- EPs® 7630–caused IL-22 production by T cells is essentially dependent on monocytes.
- IL-22 increase antimicrobial proteins (AMPs) in airway epithelium.
- EPs® 7630 might protect against airway infection by induction of AMP-inducers.

**Electronic supplementary material:**

The online version of this article (10.1007/s00109-020-01970-3) contains supplementary material, which is available to authorized users.

## Introduction

Root preparations of *Pelargonium sidoides*, a medical herb belonging to the *Geraniaceae* family, have a long history of traditional use mainly for the treatment of diarrhea, intestinal colic, anemia, weakness, and complications related to dysentery and in rare cases also for respiratory tract infections including tuberculosis in southern Africa [1]. More than 100 years ago, its commercialization started in the UK resulting in the development of the standardized ethanolic root extract EPs® 7630 (Umckaloabo®, ISO Arzneimittel, Ettlingen, Germany), which is approved for the treatment of acute bronchitis in Germany. Clinical effectiveness of EPs® 7630 has also been suggested for other indications such as rhinosinusitis, tonsillopharyngitis, and common cold [2–4]. Moreover, in COPD patients, EPs® 7630 was observed to prolong the duration between exacerbations and to reduce their overall frequency [5]. Reduced asthma attack and cough frequency was also reported for EPs® 7630–treated compared with untreated asthmatic children [6].

EPs® 7630 contains several active substances including highly oxygenated coumarin derivatives (e.g., umckalin) and, most prominently, oligomeric proanthocyanidins (polyphenols), which make up about 40% of the total dry mass [7, 8]. *Pelargonium* preparations were reported to strengthen the defense mechanisms of the body against different types of pathogens.

Indeed, EPs® 7630–mediated effects include anti-infective properties that support limitation of viral and bacterial infections without microbial resistances promoting potential [9]. These properties are based on mucokinetic effects [10] and the ability to counteract viral adhesion and spreading as well as bacterial adherence [9, 11–18]. Furthermore, EPs® 7630 has been shown to support the control of *Candida albicans* infection *in vitro* by increasing the oxidative burst of human phagocytes. Moreover, it enhanced the production of nitric oxide and inflammatory cytokine expression in *Leishmania major*–infected macrophages [17, 19–21]. Nevertheless, the mechanisms of action of EPs® 7630 are still poorly understood. The present study aimed at further elucidating the mechanisms of antimicrobial defense mediated by EPs® 7630.

## Materials and methods

### Preparation and properties of EPs® 7630

Dried extract of EPs® 7630 was prepared from *Pelargonium sidoides* roots using aqueous ethanol (11% w/w) as extracting agent in a 1:8–1:10 drug-to-solvent ratio. A stock solution of 3 mg/ml from the dried extract of a single batch (No. PSc2003/L01-11/SY06-041-A) which showed a very low–contaminating lipopolysaccharide content (< 200 EU/mg which is equivalent to about 20 ng/mg, assessed by Limulus amebocyte lysate (LAL) assay) was prepared as described before [22]. We have previously shown that cellular viability is not significantly altered by 30 μg/ml EPs® 7630 [22].

### Cell isolation and culture

Human peripheral blood mononuclear cells (PBMCs) were isolated from venous blood of healthy donors by density gradient centrifugation using Ficoll (Biochrom) as previously described [23].

The influence of EPs® 7630 on the cytokine production of PBMCs was investigated in different settings: For the kinetic study, PBMCs were stimulated with 3 to 10 μg/ml EPs® 7630, 100 ng/ml LPS (*Escherichia coli* 0127:B8 lipopolysaccharide) or were left without stimulation (control) for 4 h, 24, 48, 72, and 96 h. To study the concentration dependency of the EPs® 7630 effects, PBMCs were stimulated with increasing EPs® 7630 concentrations ranging from 0 to 3 μg/ml for 48 h.

In a further setting, PBMCs were first cultured with increasing EPs® 7630 concentrations (ranging from 0 to 3 μg/ml) only. After 24 h, anti-CD3 (Orthoclone, Janssen-Cilag) and anti-CD28 (R&D Systems) antibodies (1 μg/ml each), a cytokine mixture containing IL-1β, IL-2, and IL-12 (10 ng/ml each, R&D Systems) or control medium was added for another 24 h.

To investigate the mechanisms of EPs® 7630 effects, CD4^+^ memory T cells and monocytes were purified from PBMCs by MACS system–based negative selection using the Memory CD4^+^ T cell isolation kit and the Monocyte isolation kit II (Miltenyi Biotec), respectively, as described previously [22, 24].

In the first setting, CD4^+^ memory T cells were cultured for 48 h in the absence (control) or presence of EPs® 7630 (0–10 μg/ml). Additionally, cells were stimulated with anti-CD3/CD28 antibody–coated Dynabeads (Thermo Fisher Scientific, cell bead ratio 1:1) or were left unstimulated for the last 24 h of culture.

In the second setting, CD4^+^ memory T cells were cultured for 72 h in the presence of supernatants (25% dilution with culture medium) obtained from monocytes after a 24-h culture period with 10 μg/ml EPs® 7630 or medium (control supernatant, 25% dilution with culture medium). In a further setting, CD4^+^ memory T cells and autologous monocytes were cultured alone or co-cultured with or without (transwell system; Costar) enabled cell-cell contact (T cell/monocyte ratio: 2:1) in the absence (control) or presence of 10 μg/ml EPs® 7630 for 72 h. Furthermore, separate cultures of CD4^+^ memory T cells and autologous monocytes were each pretreated or not (control) with 10 μg/ml EPs® 7630 for 24 h and subsequently washed, reseeded, and co-cultured (T cell/monocyte ratio: 2:1) for further 72 h without any addition of stimuli.

Inhibition of cytokine effects was investigated in 72-h cultures of PBMCs using 1.5 μg IL-1RA (R&D Sytems), 3 μg/ml anti-IL-23p19 antibodies (Tremfya™, Janssen-Cilag) or a combination thereof.

All immune cell cultures described above were performed using RPMI culture medium (tested for very low endotoxin content), supplemented with 10% fetal bovine serum and 2 mM L-Glutamin (Biochrom). In all groups that served as control for EPs® 7630–stimulated groups, ethanol diluted in RPMI medium was used as solvent control (0.01% ethanol for 3 μg/ml EPs® 7630 groups; 0.033% ethanol for 10 μg/ml EPs® 7630 groups). All blood samples were approved by the clinical institutional review board of the Charité Universitätsmedizin Berlin, and written informed consent was obtained from all participants. The study was conducted according to the Declaration of Helsinki Principles.

A549 human lung epithelial cells were obtained from DSMZ (Deutsche Sammlung von Mikroorganismen und Zellkulturen, Braunschweig, Germany) and cultured in DMEM supplemented with 10% fetal bovine serum and 2 mM L-Glutamin (both from Biochrom). To test the influence of IL-22 compared to IL-17 and IFN-γ on the expression of antimicrobial peptides (AMPs), A549 cells were pre-cultured for 24 h with or without EPs® 7630 and treated afterwards with 10 ng/ml IL-22, 200 ng/ml IL-22BP, 10 ng/ml IL-17A, 10 ng/ml IFN-γ, combinations thereof, or they were left untreated (control) for 48 h.

### Flow cytometry–based analyses

The purity of isolated monocytes and memory CD4^+^ T cells was assessed by flow cytometry as described previously [22]. The mean (± SEM) purity of isolated monocytes and CD4^+^ memory T cells was 91.31 ± 1.13% and 96.62 ± 0.41%, respectively.

To characterize EPs® 7630–dependent IL-22 producers, an IL-22-specific secretion assay (Miltenyi Biotec) was performed using PBMCs according to the manufacturer’s protocol. Briefly, PBMCs were cultured in RPMI in the absence (solvent control) or presence of 10 μg/ml EPs® 7630 for 72 h followed by labeling with IL-22 catch reagent. Subsequently, cells were cultured for 3 h in the presence of 10 μg/ml EPs® 7630 under slow continuous rotation using a MACSmix device (Miltenyi Biotec). Afterwards, cells were labeled using a biotinylated IL-22 detection antibody followed by a phycoerythrin-coupled anti-Biotin antibody. All data acquisitions and analyses were performed using a FACSCalibur device and Cell-Quest software (BD Biosciences).

### Mice

Male BALB/c mice at an age of 14 weeks were i.p. injected with 1 μg recombinant murine IL-22 (R&D Systems) or a respective volume of PBS (control). After 1, 3, 24, 48, and 72 h past injection, mice were sacrificed, and lung tissue was harvested and snap-frozen for later qPCR analysis. Lung tissue from sacrificed mice that did not receive any i.p. injection served as control (0 h value). All experimental protocols have been approved by the regional authorities (Landesamt für Gesundheit und Soziales) and were conducted according to the German Animal Protection Law, as well as provisions on labor, health, and technical safety.

### ELISA

Quantification of cytokines in cell culture supernatants was performed by ELISA according to the manufacturer’s recommendation. All detection kits were purchased from R&D systems.

### RT-qPCR

Homogenization of murine lung tissue, isolation of cellular RNA from these tissues as well as from cultured A549 cells, and quantitative PCR analysis on reverse-transcribed mRNA (RT-qPCR) were performed as described previously [25]. For the quantification of S100A9, LCN2, and MX1 mRNA levels, ready-to-use systems, purchased from Thermo Fisher Scientific, were used, whereby the quantification of HPRT mRNA was included for normalization of data. All samples were analyzed in triplicates using ABI Prism 7700 Sequence Detection System or the Stepone plus system and associated software (Applied Biosystems, Darmstadt, Germany).

### Statistical analyses

Statistical analysis was performed using SPSS software (IBM). Testing for possible differences between treatment groups was performed using Wilcoxon matched-pairs signed-rank test. A *p* value of < 0.05 was considered to indicate significance.

## Results

### EPs® 7630 provokes production of lymphocytic cytokines in human immune cells

Lymphocytes of both the adaptive (T cells) and innate (e.g., innate lymphoid cells, ILCs) immune system are known to play a crucial role in the antimicrobial host defense of epithelia through production of mediators, such as IL-22, IL-17, and IFN-γ [26–28]. To shed further light on mechanisms underlying the anti-infectious action of Pelargonium-derived phytomedicals, we asked whether EPs® 7630, a standardized *Pelargonium sidoides* root extract, is able to induce the production of those cytokines by human lymphocytic cells.

**Fig. 1:**
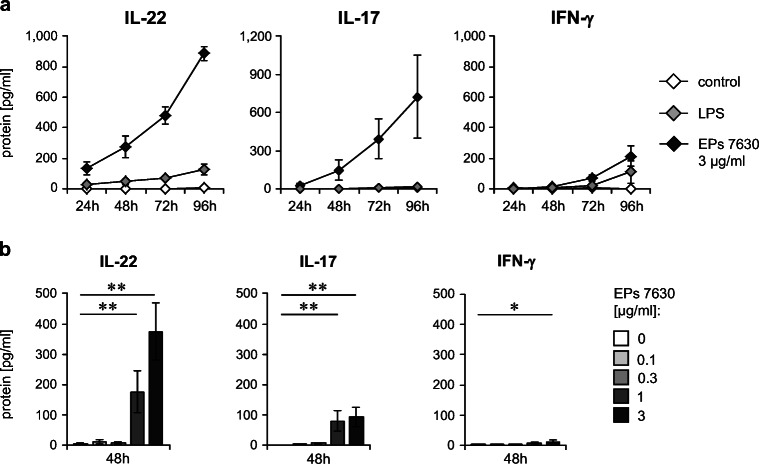
EPs® 7630 stimulates IL-22 and IL-17 secretion of human PBMCs. **a** Human PBMCs were stimulated in a kinetic approach with 3 μg/ml EPs® 7630, 100 ng/ml LPS, or were left untreated as indicated. **b** Dose-response analysis was performed by treatment of human PBMCs with increasing concentrations (0–3 μg/ml, as indicated) of EPs® 7630 for 48 h. Quantification of IL-22, IL-17, and IFN-γ in culture supernatants was performed by ELISA. Data from 3 (**a**) or 12 (**b**) independent experiments are given as mean ± SEM. Significant differences among treatment groups are indicated (*p < 0.05; **p < 0.01, Wilcoxon matched-pairs signed-rank test)

We addressed this question by first analyzing a kinetic *in vitro* approach for up to 96 h using human immune cells stimulated with 3 μg/ml of EPs® 7630. Indeed, EPs® 7630 was able to time-dependently induce the lymphocytic cytokines IL-22, IL-17, and IFN-γ (Fig. 1a). This induction was most evident for IL-22 that was detectable already after 24 h of EPs® 7630 stimulation. In contrast, induction of IL-17 and, in particular, of IFN-γ was much less pronounced and considerably delayed, starting 48 h after stimulation. Interestingly, compared with the bacterial component lipopolysaccharide (LPS), an indirectly acting inducer of these cytokines [29], EPs® 7630 was much more potent in terms of lymphocyte cytokine induction (Fig. 1a). A closer look at the concentration dependency of the cytokine-inducing effect of EPs® 7630 during a 48-h stimulation period revealed an effectivity of the root extract starting from 1 μg/ml (Fig. 1b).

### EPs® 7630 selectively strengthens IL-22 and IL-17 production of activated T cells

Next, we aimed at gaining insights into the cytokine-inducing effects of EPs® 7630 in the context of immune activation according to the frequent use of this drug in clinical practice, i.e., as infection preventive measure. In the first step, we investigated the effect of EPs® 7630 pretreatment on the activation of lymphocytes. PBMC cultures were treated with the root extract for 24 h followed by addition of T cell–stimulating anti-CD3 and anti-CD28 antibodies for a further 24-h period.

**Fig. 2:**
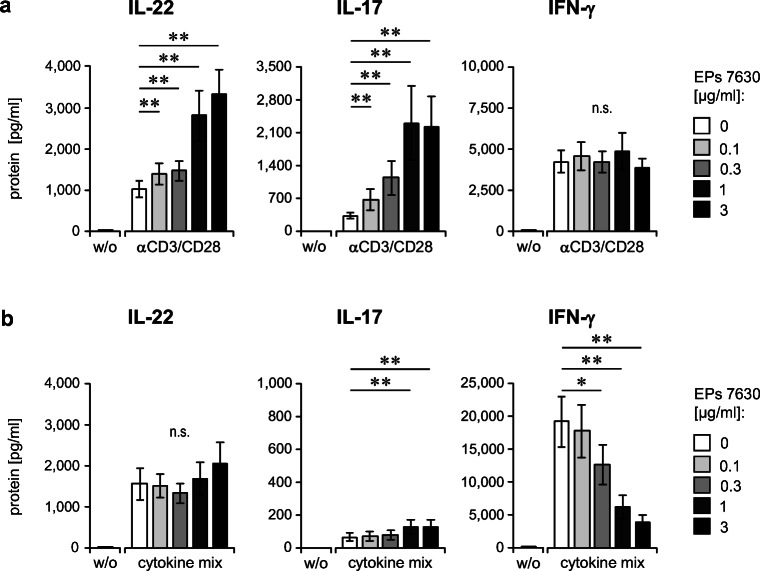
EPs® 7630 pretreatment modulates the cytokine production of activated lymphocytes. **a**, **b** Human PBMCs were pretreated with different concentrations of EPs® 7630 as indicated for 24 h. Afterwards, activators of T cells (anti-CD3/anti-CD28 Abs) (**a**) and innate lymphoid cells (cytokine mixture composed of IL-1β, IL-2, and IL-12) (**b**) were added for further 24 h. Quantification of IL-17, IL-22 and IFN-γ in culture supernatants was performed by ELISA. Data from 12 donors per group are given as mean ± SEM. Significant differences are indicated (*p < 0.05; **p < 0.01, Wilcoxon matched-pairs signed-rank test)

As demonstrated in Fig. 2a, EPs® 7630 induced a clear, concentration-dependent increase of IL-22 and IL17, starting from a concentration of as low as 0.1 μg/ml. At the highest concentration (3 μg/ml), EPs® 7630 provoked an increase in IL-22 and IL-17 production of 3.9 ± 0.5- and 7.3 ± 2.0-fold, respectively, compared with stimulated cultures without EPs® 7630 pretreatment (Fig. 2a). In contrast, EPs® 7630 did not influence the production of IFN-γ by activated T cells (Fig. 2a), excluding its action as a general amplifier of T cell cytokine responses.

### EPs® 7630 has no effect on IL-22 and IL-17 production by innate lymphoid cells but strongly limits their IFN-γ production capacity

Besides activated T cells, ILCs of the innate immune repertoire also play an essential role in the host defense against infections by production of IL-22 and IL-17 [28]. Therefore, we next investigated the effect of EPs® 7630 pretreatment on the activation of these cells, which can be achieved by stimulation with specific cytokines. Thus, PBMC cultures were treated with EPs® 7630 for 48 h with addition of IL-1β, IL-2, and IL-12 for the last 24 h of culture.

Interestingly, in the context of cytokine-stimulation, EPs® 7630 had no effect on IL-22 and only a minimal enhancing effect on IL-17 production. However, it strongly and concentration-dependently inhibited IFN-γ production, with an average fold inhibition at the highest concentration of EPs® 7630 (3 μg/ml) of 6.0 ± 0.7 compared with cytokine-stimulated cultures without EPs® 7630 pretreatment (Fig. 2b). These data hint to T cells as the source of IL-22 and also IL-17 in EPs® 7630–treated or pretreated immune cells.

### The EPs® 7630–induced cytokine IL-22 increases the antimicrobial airway defense

In previous studies, we have demonstrated that IL-22 is a potent inducer of the cutaneous antimicrobial defense [30, 31], with IL-17 frequently enhancing this IL-22 effect [32, 33]. As EPs® 7630 is used for the clinical indication of airway infections, the relevance of EPs® 7630–induced IL-22 was tested using respective *in vitro* and *in vivo* models.

**Fig. 3:**
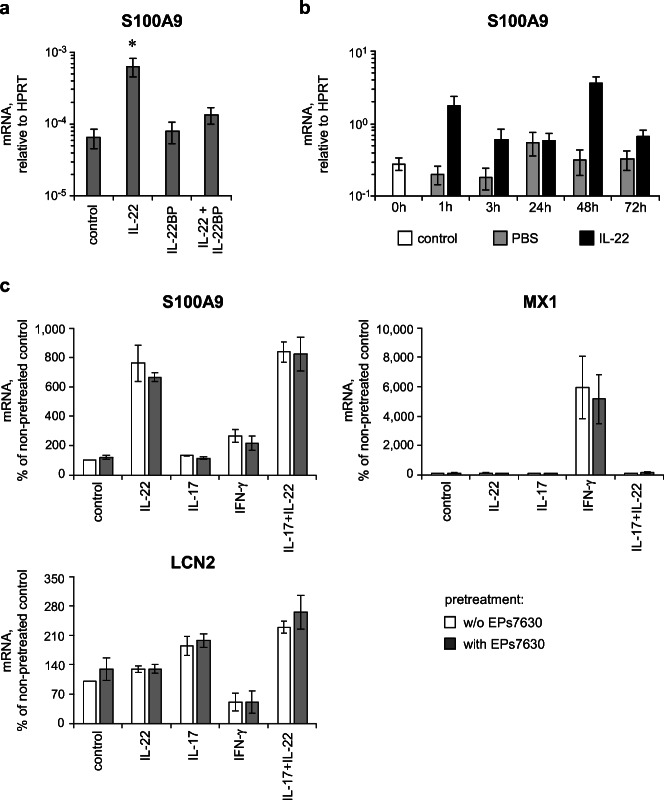
IL-22 strengthens the antibacterial defense of airway epithelial cells. **a** A549 human lung epithelial cells were cultured in the presence or absence (control) of IL-22, its inhibitor IL-22BP, or a combination of IL-22 and IL-22BP for 48 h. **b** BALB/c mice were i.p. injected with PBS (control) or IL-22. At the indicated time points after injection, mice were sacrificed and lung tissue was taken for analysis of S100A9 expression by RT-qPCR. Data of 4 (0 h control and PBS) or 3 (IL-22) mice per group are given as mean ± SEM. **c** A549 human lung epithelial cells were pretreated or not with 3 μg/ml EPs® 7630 for 24 h followed by stimulation with IL-22, IL-17A, IFN-γ, or the combination of IL-17A and IL-22 for 48 h or were left unstimulated (control). **a**, **c** Expression of S100A9, LCN2, and MX1 was analyzed by RT-qPCR. Data of 7 (**a**) or 3–4 (**c**) independent experiments are given as mean ± SEM. Significant differences among treatment groups are indicated (*p < 0.05, Wilcoxon matched-pairs signed-rank test)

As demonstrated in Fig. 3a, IL-22 stimulation of cultured airway epithelial A549 cells strongly enhanced the production of the AMP S100A9, a known IL-22 downstream target [31]. This effect was found to be specific, as blocking of IL-22 action by its natural soluble inhibitory receptor, IL-22 binding protein (IL-22BP) [34–37], abrogated the IL-22-caused induction of S100A9.

In line with these data, *in vivo* intraperitoneal application of recombinant murine IL-22 provoked a strong increase in pulmonary S100A9 expression compared with PBS-treated mice (Fig. 3b). Notably, in consideration of the limited half-life of IL-22 and the single application mode, the S100A9 inducing effect was quite long-lasting, being still measurable after 72 h (Fig. 3b). Of note, EPs® 7630 did not have an influence itself or modulated the IL-22-induced S100A9 or IL-17-induced LCN2 expression in A549 cells (Fig. 3c). Furthermore, IL-22 did not induce the expression of the antiviral protein MX1 in contrast to its known inducer IFN-γ, confirming the target specificity of IL-22 in this setting. However, in contrast to IL-22, IFN-γ did not modulate expression of S100A9.

### EPs® 7630–induced IL-22 production by T cells is essentially dependent on monocytes

**Fig. 4:**
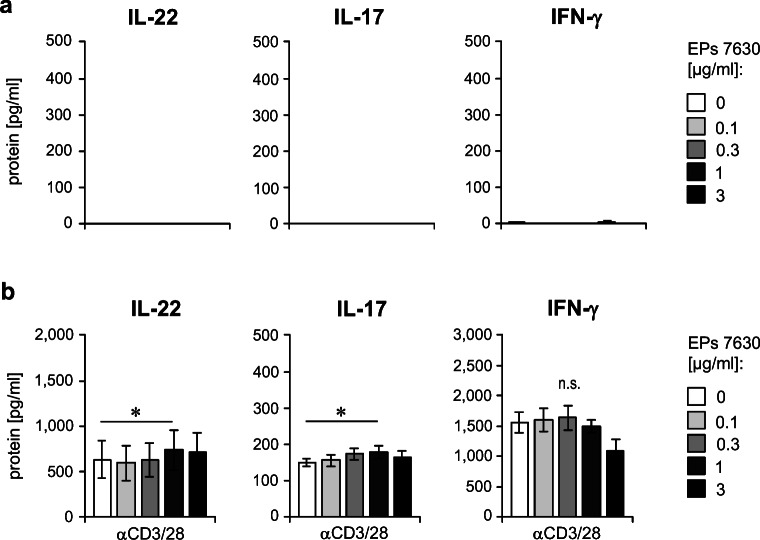
CD4^+^ memory T cells are no direct targets of EPs® 7630. **a** CD4^+^ memory T cells were isolated from human PBMCs by magnetic labeling–based cell sorting and cultured for 48 h in the absence (control) or presence of EPs® 7630 (0–10 μg/ml) as indicated. **b** CD4^+^ memory T cells were stimulated as described in **a** and were further activated after 24 h of culture by anti-CD3/CD28 antibodies (Dynabeads) for another 24 h. **a**, **b** Culture supernatants were analyzed for IL-22, IL-17, and IFN-γ level by ELISA. Data from 5 independent experiments are given as mean ± SEM. Significant differences among treatment groups are indicated (*p < 0.05, Wilcoxon matched-pairs signed-rank test)

Next, we aimed to study the EPs**®** 7630–induced cytokine production by T cells in more detail. For this purpose, we purified CD4^+^ memory T cells from freshly obtained PBMC. When stimulating these cells with EPs® 7630 using the protocol applied for PBMC cultures before (Fig. 1a), we surprisingly did not detect any IL-22, IL-17, and IFN-γ in respective culture supernatants (Fig. 4a). Furthermore, EPs® 7630 had no relevant influence on the cytokine production of CD4^+^ memory T cells stimulated *via* CD3/CD28 (Fig. 4b). These data raised the hypothesis that other immune cell types or their mediators might be involved in the effects of EPs® 7630 on T cell cytokine production.

**Fig. 5:**
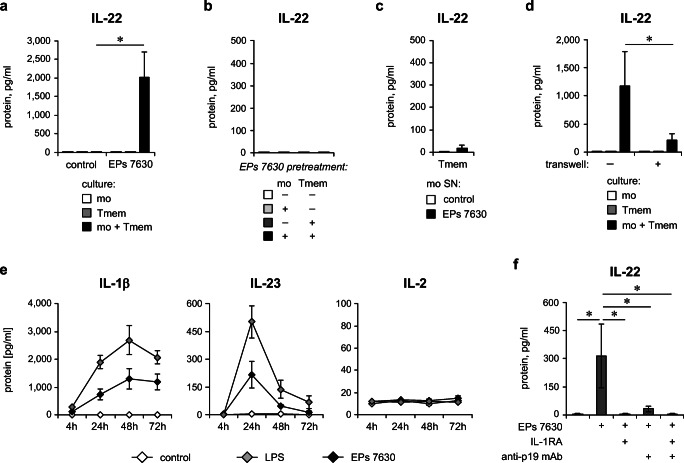
Monocytes play a key role in EPs® 7630–induced IL-22 production by T cells. **a** CD4^+^ memory T cells and autologous monocytes were co-cultured or cultured alone for 72 h in the absence (control) or presence of 10 μg/ml EPs® 7630 (EPs® 7630). **b** CD4^+^ memory T cells and autologous monocytes were pretreated in separate cultures with EPs® 7630 (10 μg/ml) or medium with solvent for 24 h. Afterwards, CD4^+^ memory T cells and monocytes were washed and co-cultured as indicated for 72 h without further EPs® 7630 stimulation. **c** CD4^+^ memory T cells were cultured for 72 h in the presence of supernatant (SN) obtained from cultures of EPs® 7630–stimulated (10 μg/ml) monocytes. **d** CD4^+^ memory T cells and autologous monocytes were co-cultured with (no transwell) or without enabled cell-cell contact (transwell) or were cultured separately for 72 h in the presence of 10 μg/ml EPs® 7630. **a**–**d** Human CD4^+^ memory T cells and autologous monocytes were each isolated by magnetic labeling–based cell sorting. Quantification of IL-22 in culture supernatants was carried out by ELISA. **e** Human PBMCs were stimulated or not (solvent control) in a kinetic approach with 10 μg/ml EPs® 7630 or 100 ng/ml LPS or were left without stimulation up to 72 h. Quantification of IL-1β, IL-23, and IL-2 levels in culture supernatant was performed by ELISA. **f** Human PBMCs were stimulated or not (solvent control) with 3 μg/ml EPs® 7630, in the presence of 1.5 μg/ml IL-1RA, 3 μg/ml anti-IL-23p19 antibody, or a combination thereof for 72 h. Quantification of IL-22 in culture supernatants was carried out by ELISA. Data from 6 (**a**), 2 (**b**), 4 (**c**), 5 (**d**) 3–4 (**e**), and 5 (**f**) independent experiments are given as mean ± SEM. Significant differences between treatment groups are indicated (*p < 0.05, Wilcoxon matched-pairs signed-rank test). Tmem: CD4^+^ memory T cells; mo: monocytes

We therefore performed EPs® 7630 stimulation of CD4^+^ memory T cells co-cultured with autologous monocytes. As demonstrated in Fig. 5a, EPs® 7630 in fact provoked a strong production of IL-22, whereas this effect was absent in the separately cultured cell populations. Next, we tested whether cell-cell contact is sufficient for EPs® 7630–induced IL-22 production in T cells. The use of EPs® 7630–pretreated monocytes and autologous CD4^+^ memory T cells, which were subsequently co-cultured without EPs® 7630, however, did not result in IL-22 production (Fig. 5b). These data imply that cell-cell contact with monocytes alone is insufficient or even not relevant to provoke IL-22 production in T cells and suggest that soluble mediators produced by monocytes might play a role here. Therefore, we investigated whether supernatants obtained from EPs® 7630–stimulated monocytes would provoke IL-22 production by CD4^+^ memory T cells. Surprisingly, the transfer of monocyte culture supernatant had no relevant effect on the IL-22 production by CD4^+^ memory T cells either (Fig. 5c). These data show that cytokines produced by monocytes and the presence of EPs® 7630 alone are also not sufficient to induce IL-22 in T cells. We therefore hypothesized that a dependency on soluble mediators as well as direct cell-cell contact with monocytes in the presence of EPs® 7630 might be necessary for EPs® 7630–induced IL-22 production by CD4^+^ memory T cells. To test this hypothesis, we compared co-cultures of CD4^+^ memory T cells and autologous monocytes in the presence of EPs® 7630 with or without the use of a transwell culture system. As demonstrated in Fig. 5d, the EPs® 7630–induced IL-22 production was prevented by 70.8 ± 15.5% in transwell co-culture conditions (contact between monocytes and T cells is impossible) compared with co-culture with enabled cell-cell contact. These data indicate that both soluble mediators produced by monocytes and direct contact with these cells are necessary for the EPs® 7630–induced IL-22 production by CD4^+^ T cells.

To identify the soluble mediators involved in IL-22 induction in T cells by EPs® 7630, we next analyzed the supernatants of EPs® 7630–stimulated PBMC cultures for the presence of T17/T22 lineage–supporting cytokines. In fact, a strong upregulation of IL-1β and IL-23 but not IL-2 was detected in culture supernatants early after EPs® 7630 stimulation (Fig. 5e). Importantly, blocking of IL-1 by IL-1 receptor antagonist and IL-23 by anti-IL-23p19 antibody (guselkumab) strongly reduced IL-22 production in the co-culture system of CD4^+^ memory T cells and autologous monocytes with enabled cell-cell contact (Fig. 5f). Although less prominently induced by EPs® 7630, expression of IL-17 was observed to underlie regulatory mechanisms similar to those detected for IL-22 (Fig. S1).

### EPs® 7630 induces IL-22 secretion by different CD4^+^ memory T cell subsets

**Fig. 6:**
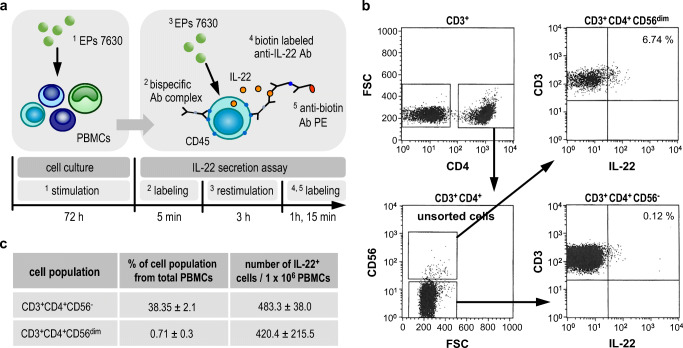
EPs® 7630 induces IL-22 secretion by different CD4^+^ memory T cell subsets. **a**–**c** Human PBMCs were stimulated or not (solvent control) with 10 μg/ml EPs® 7630 for 72 h as indicated. Afterwards, an IL-22-specific secretion assay and surface marker staining was performed followed by flow cytometry–based analysis. **a** Schematic overview of the experimental setting of IL-22-specific secretion assay. **b** Data from one representative out of three independent experiments are given. **c** Data from 3 independent experiments are given as mean ± SEM

Within the CD4^+^ memory T cell (CD4^+^ CD45RO^+^ T cell) compartment, classical CD4^+^ memory T cells (CD3^+^CD4^+^CD56^−^) as well as CD4^+^ NKT cells are described as IL-22 producers [38–44]. Using a novel IL-22-specific secretion assay (Fig. 6a), we therefore analyzed the IL-22 production capacity of CD4^+^ T cells in the context of EPs® 7630 stimulation. As shown in Fig. 6b and Fig. S2, EPs® 7630 stimulation provoked IL-22 production in CD3^+^CD4^+^CD56^−^ cells as well as in a cell population showing an NKT-like phenotype (CD3^+^CD4^+^CD56^dim^). Although the frequency of IL-22 producers was found to be less pronounced among CD3^+^CD4^+^CD56^−^ compared with CD3^+^CD4^+^CD56^dim^ cells, the considerably higher frequency of CD3^+^CD4^+^CD56^−^ cells among blood immune cells implies an equal biological relevance of both populations for the EPs® 7630–induced IL-22 production (Fig. 6c).

## Discussion

*Pelargonium* root extract EPs® 7630 is clinically used for the treatment of acute bronchitis. However, despite the long history of its medical use, the immunomodulatory effects of EPs® 7630 are still poorly understood.

In the present study, we have identified a new immunoregulatory property of EPs® 7630. In fact, we found that EPs® 7630 selectively enhances adaptive T22 and T17 immune responses by increasing the constitutive and activation-dependent T cell production of IL-22 and IL-17. Both of these cytokines are key players in the host defense to combat bacterial and fungal infections through induction of AMPs [30–33]. Accordingly, we could show that IL-22 strongly enhanced the expression of S100A9 in lung epithelial cells *in vitro*. EPs7630 thereby neither had an influence itself nor influenced the IL-22-induced S100A9 expression in A549 cells. Furthermore, a pulmonary increase of S100A9 expression was observed *in vivo* after a single application of IL-22. S100A9 is a member of the S100 family of proteins that exerts its antimicrobial effect, which is directed against bacteria and fungi, through metal ion sequestration [45].

Interestingly, the IL-17 and IL-22-inducing effect by EPs® 7630 was not clearly observed with respect to innate lymphoid cells. In these cells, however, EPs® 7630 had a strong inhibitory effect on IFN-γ production. These data hint to a role of EPs® 7630 in limiting IFN-γ-dependent tissue damage during EPs® 7630 therapy of airway infection.

The course and duration of viral bronchitis is frequently complicated by secondary bacterial infections (superinfections), which require antibiotic treatment in severe cases. However, antibiotic treatments also involve undesired effects to the patient’s microbiome, not only affecting the gut but also cutaneous and mucosal outer body barriers [46]. Furthermore, antibiotic use entails the risk of developing resistances by the microbes against the drug. A relevant factor promoting superinfections is the virus-induced downregulation of AMPs [47]. Notably, by upregulating the AMP-inducers IL-22 and IL-17, EPs® 7630 might counteract this AMP deficiency, suggesting a protective role of EPs® 7630 against superinfections of respective patients. However, future *in vivo* studies are needed to prove this hypothesis.

We found monocytes to be an essential factor for mediating the EPs® 7630 effects on T cell responses. This observation is in line with our previous study showing that monocytes are directly targeted by EPs® 7630, resulting in MAP kinase activation [22]. We now could show that induction of IL-22 in T cells is essentially dependent on three factors: a direct cell-cell contact with monocytes, the permanent presence of EPs® 7630, and soluble mediators produced by monocytes. By specifically blocking cytokine activity, we indeed found IL-1 and IL-23 to be those mediators essential for IL-22 induction by T cells.

There are currently no clear data regarding the bioavailability of EPs® 7630. For anthocyanins, a group of polyphenols that also display the main constituents of EPs® 7630, a bioavailability of ~ 1–12% was observed in several studies [48]. Assuming a comparable bioavailability for EPs® 7630, the dosages of 0.1–10 μg/ml used in this study are within the estimated range of drug serum levels after ingestion of a single recommended dose of 20 mg EPs® 7630 [48]. Overall, our study suggests a new protective role for EPs® 7630 against bacterial airway superinfections by induction of the AMP-inducers IL-22 and IL-17.

## Electronic supplementary material

ESM 1 (DOCX 153 kb)
