# Supplementary material for: The herbal extract EPs® 7630 increases the antimicrobial airway defense through monocyte-dependent induction of IL-22 in T cells
Source: J Mol Med (Berl). 2020 Sep 19;98(10):1493–503. doi: 10.1007/s00109-020-01970-3 (PMC7524690; doi:10.1007/s00109-020-01970-3)
Supplement: Supplementary file 1 — (DOCX 153 kb) [file 109_2020_1970_MOESM1_ESM.docx]

**The herbal extract EPs^®^ 7630 increases the antimicrobial airway defense through monocyte-dependent induction of IL-22 in T cells**

Katrin Witte^1,2^, Egon Koch^3^, Hans-Dieter Volk^2,4^, Kerstin Wolk^1,2,5^, Robert Sabat^1,5^

^1^ Interdisciplinary Group of Molecular Immunopathology, Dermatology/Medical Immunology, Charité - Universitätsmedizin Berlin, Germany;

^2^ BIH Center for Regenerative Therapies, Charité - University Medicine, Berlin, Germany

^3^ Dr. Willmar Schwabe GmbH & Co. KG, Karlsruhe, Germany; present address: Am Giessbach 11a, 76229 Karlsruhe, Germany

^4^ Institute of Medical Immunology, Charité - Universitätsmedizin Berlin, Germany

^5^ Psoriasis Research and Treatment Center, Charité - Universitätsmedizin Berlin, Germany

**Correspondence:**

Robert Sabat, Psoriasis Research and Treatment Center, Department of Dermatology and Medical Immunology, Universitätsmedizin Berlin, Charitéplatz 1, Berlin D-10117, Germany. E-mail: [robert.sabat@charite.de](mailto:robert.sabat@charite.de)

**Supplementary material**

**Supplementary figures: Figure S1-S2**

**Fig. S1 Regulation of EPs**^®^ **7630-induced IL-17 production in immune cells.** a CD4^+^ memory T cells and autologous monocytes were co-cultured or cultured alone for 72 h in the absence (control) or presence of 10 µg/ml EPs® 7630 (EPs® 7630). b CD4^+^ memory T cells and monocytes were pretreated in separate cultures with EPs® 7630 (10 µg/ml) or medium with solvent for 24 h. Afterwards, CD4^+^ memory T cells and monocytes were washed and co-cultured as indicated for 72 h without further EPs® 7630 stimulation. c CD4^+^ memory T cells were cultured for 72 h in the presence of supernatant (SN) obtained from cultures of EPs® 7630-stimulated (10 µg/ml) monocytes. d CD4^+^ memory T cells and autologous monocytes were co-cultured with (no transwell) or without enabled cell-cell contact (transwell) or were cultured separately for 72 h in the presence of 10 µg/ml EPs® 7630. (a-d) Human CD4^+^ memory T cells and autologous monocytes were each isolated by magnetic labeling-based cell sorting. Quantification of IL-17 in culture supernatants was carried out by ELISA. f Human PBMCs, isolated from the blood of healthy donors, were stimulated or not (solvent control) with 3 µg/ml EPs® 7630, in the presence of 1.5 µg/ml IL-1RA, 3 µg/ml anti-IL-23p19 antibody or a combination thereof for 72 h. Quantification of IL-17 in culture supernatants was carried out by ELISA. Data from 6 (a), 2 (b), 4 (c) and 5 (d, e) independent experiments are given as mean ± SEM. Significant differences between treatment groups are indicated (* p<0.05, Wilcoxon matched-pairs signed-rank test). Tmem: CD4^+^ memory T cells; mo: monocytes.

**Fig. S2 IL-22 secretion assay performed using solvent control treated PBMCs. a-b** PBMCs, isolated from the blood of healthy donors, were stimulated or not (solvent control) with 10 µg/ml EPs^®^ 7630 for 72 h as indicated. Afterwards an IL-22-specific secretion assay and surface marker staining was performed followed by flow cytometry based analysis. **a** Data from one representative out of three independent experiments are given. **b** Data from 3 independent experiments are given as mean ± SEM.
